# Nanotechnology's Applications and Potential in Various Fields

**DOI:** 10.7759/cureus.59234

**Published:** 2024-04-28

**Authors:** Asmith K Soni, Roshan K Jha

**Affiliations:** 1 Medical Education, Jawaharlal Nehru Medical College, Datta Meghe Institute of Higher Education and Research, Wardha, IND; 2 Biochemistry, Jawaharlal Nehru Medical College, Datta Meghe Institute of Higher Education and Research, Wardha, IND

**Keywords:** gene delivery, biomedical applications, nanomedicine, nanoparticles, nanotechnology

## Abstract

Since ancient times, several sorts of nanoparticles have been employed in the quickly expanding field of nanotechnology. These features include size, shape, and chemical as well as physical properties. Because of their small size and huge surface area, carbon-based nanoparticles, including fullerenes, carbon nanotubes, graphene, graphene oxide, and carbon-based quantum dots, have attracted a lot of attention in a variety of sectors, including biomedical applications. Lipid bilayers form the spherical vesicles known as liposomes. Magnetic resonance imaging (MRI) contrast agents are iron oxide nanoparticles. These materials are perfect for drug and delivery of genes, bioimaging, and bone repair because of their remarkable mechanical, electrical, visual, and chemical properties. However, concerns about potential asbestos-related diseases have arisen due to their length-to-width aspect ratio. Ceramic nanoparticles, on the other hand, are a common material in daily life and play a crucial role in bone repair, multiscale hybridisation, and aerospace structures. These nanoparticles can enhance osseointegration and bone development by mimicking the nanocomposition and nanoscale characteristics of bone tissue and enhance osteoconductive and osteoinductive capacities. Ceramic nanoparticles, however, have the potential to generate oxidative stress, which can result in irritation of the reticuloendothelial system, cytotoxicity to the heart, liver, and lungs, as well as toxicity to the cells that are attached. Additionally, oxidative stress, cell damage, and genotoxicity might result from the generation of free radicals by ceramic nanoparticles. Metal nanoparticles exhibit linear optical properties similar to molecular systems but arise from a different physical process. Semiconductor nanocrystals (NCs) are made from various compounds, such as silicon and germanium. Polyandry nanoparticles are particles approximately 10 and 10000 nanometers (nm) in size that can contain active substances. They have applications in vaccine delivery, gene therapy, and polymer nanoparticles (nanomedicine) for therapeutic applications.

## Introduction and background

Nanotechnology insight

Nanotechnology is the controlled alteration of size and shape at the nanometer level to produce structures, devices, and systems with a minimum of one novel characteristic or property. By carefully controlling the size and manipulating the shape at the nanometer level (atomic, molecular, and macromolecular scales), nanotechnology entails imaging, modelling, measuring, designing, characterising, producing, and applying structures, devices, and systems, producing structures, devices, and structures with at least one novel/superior characteristic and property [1]. Between 1- and 100-nanometer-long objects can be studied and worked on; nanotechnology refers to research and technological advancements involving atomic, molecular, or macromolecular sizes. These objects, like "nanoparticles," acquire unique characteristics and functions that are very different from those observed at the bulk size. Numerous new areas of biological study are made possible by nanoparticles. By functioning at the very size of biomolecules, nanoparticles' unique features enable the possibility of novel interactions with intricate biological processes. Researchers from several fields may design and create nanoparticles with multiple properties which target, identify, and resolve diseases like AIDS in this quickly expanding field [2]. The frontier of the 21st century, known as nanotechnology, was created by human fantasy. To enable new applications, it includes comprehending and manipulating. Richard Zsigmondy, the 1925 Chemistry Nobel Laureate, and Richard Feynman, the 1965 Physics Nobel Laureate, initially introduced the idea of a "nanometer" in 1959. "Nanotechnology" was initially used to describe semiconductor operations as nanometers by Japanese scientist Norio Taniguchi. Following the finding of fullerenes in the 1980s, nanotechnology entered its heyday. Taniguchi's phrase and "There Is Plenty of Room at the Bottom" by Richard Feynman were both referenced in Eric Drexler's 1986 book "Engines of Creation: The Coming Era of Nanotechnology." An improved field of nanotechnology developed carbon nanotubes. The National Nanotechnology Initiative (NNI) was established in the 21st century due to President Bill Clinton's advocacy for financing and the 21st Century Nanotechnology Research and Development Act [3].

Thanks to advances in nanoscience and nanotechnology, researchers in India now have a multidisciplinary platform to solve a range of pressing problems in materials engineering, manufacturing, military, healthcare, and cosmetics. The Indian government has launched several efforts in recent years to entice young researchers to begin their work in the multidisciplinary fields of nanotechnology and related fields. Indian researchers are assisted by a variety of programmes administered by several ministries of the Indian government to build both simple and sophisticated infrastructure, develop human resources, offer scholarships, and promote awareness. The initiatives have established a common forum to draw researchers (faculty and students) from the physical, chemical, biological, mathematical, computational, and engineering sciences to collaborate internationally and exchange ideas to overcome demographic and infrastructure challenges [4]. The science and engineering behind designing, synthesising, characterising, and using objects and materials whose tightest functional organisation, in no less than one dimension, is on the nanoscale, or a billionth of a meter, is known as nanotechnology. At these scales, each molecule and communicating groups of molecules are considered about the material or device's bulk macroscopic properties because they influence the basic molecular structure, enabling control for the macro-level chemical and physical properties [5]. A gene is the primary structural and functional element of heredity. Genes are made from DNA. The components of specific genes are used to create proteins. A human gene can be as little as a few hundred DNA bases or as large as more than 200,000. The Human Genome Project, a global scientific endeavour to understand the sequencing of the human genome and identify its genes, estimates that humans contain between 20,000 and 25,000 genes [6].

Our unique genetic code is stored in a huge molecule called deoxyribonucleic acid, or DNA. Like a recipe book, it offers instructions for creating each protein in our body. The substance known as deoxyribonucleic acid, or simply DNA, makes up your genome. Four fundamental building units, or "bases," make up DNA: adenine, cytosine, guanine, and thymine. The configuration, or order, of these nucleotides creates the genome's instructions. The instructions in the genome are created by the arrangement, or configuration, of these nucleotides: a two-abandoned particle, DNA. DNA is a molecule with two strands [7]. The terms "DNA chain" and "DNA strand" are used for each chain. Between each base of the nucleotides, hydrogen bonds keep the two chains intact. For each of these chains, the terms "DNA chain" and "DNA strand" are used. Between each base of the nucleotides, hydrogen bonds hold the two chains together. A nucleotide is created when a base with nitrogen and many phosphate groups is joined to a five-carbon sugar. The base can be between adenine (A), cytosine (C), guanine (G), and thymine (T) in the case of the nucleotides in DNA, where DNA gets its name from the deoxyribose that is joined to a monophosphate group in the sugar. The nucleotides are linked together in a chain by the sugars and phosphates, acting in opposition to one another to create a "backbone" of sugar-phosphate-sugar-phosphate which produces covalent bonds [8]. Genetic engineering is the purposeful alteration of DNA molecules involving DNA recombination or nucleic acid mutation to modify a living being or collection of living things. Additionally, additional genetically engineered species, such as disease-resistant plants, have been produced using these methods [9].

Nanotechnology advancements

The process of performing genetic engineering is given below: finding and collecting the DNA from a live entity that possesses a certain trait and discovering and cloning the gene responsible for the trait. Creating genetic material with a specific pattern of expression, like transformation, involves introducing foreign DNA into an organism's genome and altering its genetic makeup, typically achieved via vectors like plasmids. Transgene it on a backdrop of luxury. The use of biology to create new products, procedures, and organisms to improve biological diversity is known as biotechnology (human health and society as a whole). The domestication of plants and animals led to the development of biotechnology, often known as biotech animals and also from the discovery of fermentation, which has been around since the dawn of civilisation [10]. As previously indicated, this is a fairly wide term that can refer to both cutting-edge laboratory methods and conventional agricultural and culinary methods that have been used for many years [11].

Beer Brewing

Beer brewing is an age-old craft that blends science and art. It starts with malted barley, water, hops, and yeast. Barley is mashed to release sugars and then boiled with hops for flavour and aroma. Fermentation follows, converting sugars into alcohol and carbonation. Patience and precision create diverse and delicious brews enjoyed worldwide [12].

Gene Therapy

Gene therapy is a novel approach to treating genetic diseases caused by faulty genes. It introduces the "missing" gene's DNA into the body's cells. For instance, individuals with the hereditary disease cystic fibrosis are deficient in a gene that codes for a chloride channel made for the lungs. In the clinical trial for gene therapy, cubed spheres of membrane carrying a functioning gene were sprayed onto patient lung cells. The spheres were then inhaled by the patient [11].

Nanotechnology is the creation, production, or application of a structure, device, or system with one or more dimensions on a scale of 100 nanometers (one billionth of a millimetre) or fewer hours or that manipulates atoms and molecules in the nanoscale [13]. The study and manipulation of materials is known as nanotechnology at the nanoscale. The "nanoscale" is concerned with dimensions from about 10 to 100 nanometers [14]. Over the past two decades, research and development have led to innovations in nanotechnology and the creation of bespoke materials with specific properties at the nanoscale. This greatly expands the materials science toolkit available to researchers, process engineers, and the industry. A lighter, stronger, more durable, and more reactive nanomaterial was created. Research has yielded materials with improved electrical conductivity and complex structures that are suitable for many different applications at the cutting edge of materials science and in many scientific disciplines [15]. There are many different types of nanotechnology, each with its uses and properties. Some examples are nanomaterials, nanoelectronics, nanooptics, nanomedicine, nano-energy, and nanorobotics [16].

The length scale of cellular activities is compatible with the microscale at which the interaction of cells and tissues with materials is often examined. However, the biomolecular signals that underpin these interactions between cells and their environment, such as proteins, enzymes, and genetic information, are often smaller than 100 nm. It is now conceivable to create nanoscale interventions on these pertinent length scales to imitate biomechanics using techniques from the physical sciences, thanks to the development of nanotechnology in medicine. Development of devices, medication delivery, biomaterial design, and detection and diagnostics are a few potential uses for nanoscale improvements. Nanoscale developments may have a considerable influence on our knowledge of biological interactions and the creation of new therapies despite their seeming insignificance [17].

## Review

Search methodology

**Figure 1:**
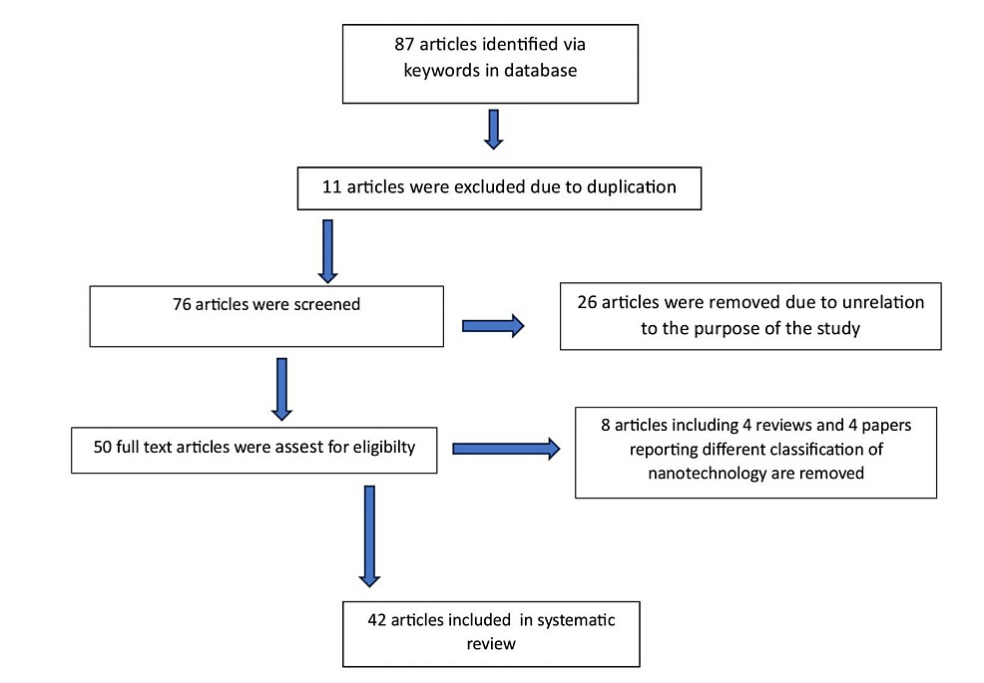
PRISMA flowchart PRISMA: Preferred Reporting Items for Systematic Reviews and Meta-Analyses

We undertook a systemic search through PubMed and Google Scholar from April to May 2023 using keywords such as "gene delivery," "biomedical applications," "nanomedicine," "nanoparticles," and "nanotechnology." The publication date of the article ranges from 1998 to 2022. One reviewer independently checked the papers retrieved based on title and abstract against the inclusion criteria before moving on to the full text. Finally, 42 articles were included in the review. Figure 1 shows the literature search strategy. 

Nanotechnology

Nanotechnology is the manipulation and control of matter at the atomic or molecular level, resulting in the development of new materials, structures, and devices with different properties and functions. It has multiple applications in medicine, electronics, materials science, and other fields, enabling new approaches to a wide range of difficulties and opportunities. Nanotechnology is a new and vital field of science. Many nanoparticles have been in use for centuries. Still, many people don't know about it. The subject of nanotechnology is the creation and application of materials having nanoscale dimensions in various facets of life. Due to their small size, nanoparticles have a high surface-to-volume ratio. Developing novel techniques for producing new goods, formulating novel materials and chemicals, and replacing present-day equipment with more efficient equipment are some of the main applications of nanotechnology, which is a young and developing field. Nanotechnology contributes to environmental remediation by developing nanoparticles for effective pollution removal from air, water, and soil. It also reduces material and energy consumption by developing more potent and lighter materials, as well as energy-efficient technologies such as nanoscale transistors and improved solar panels, promoting sustainability and resource conservation [18]. What is nanoscale? Nanoscale is one billionth of a meter (10^-9^), similar to the size of an atom and molecule. Zinc oxide or titanium dioxide nanoparticles are found in several sunscreens. Although these nanoparticles are transparent on the skin, they provide great UV protection. Effective UV protection is made possible by the employment of nanoscale particles in a clear sunscreen that is aesthetically pleasing. The sunscreen market has undergone a technological revolution, offering consumers greater convenience and protection [19].

Types of nanoparticles

Processing materials at the nanoscale, a billion times smaller than a meter, represents a game changer in technological development. In fact, "nanotechnology" refers to any nanoscale technology that has practical applications. Nanotechnology is the study and application of chemical, physical, and biological components from individual atoms and molecules to very small sizes and the assimilation of the resulting nanomaterials into larger systems. Nanotechnology has the potential to transform our approach to global crises completely. It allows the development of advanced materials for resource conservation, clean energy generation, and water purification. The innovative solutions provided by nanotechnology, ranging from more efficient solar cells to novel drug delivery systems, offer hope for addressing pressing global issues such as climate change, energy shortages, and healthcare needs. The discovery and application of carbon nanomaterials have enabled several new research projects in nanotechnology, biosensors, and bioelectronics. While bioelectronics incorporates biological elements into electronic equipment to improve medical diagnosis and healthcare technology, biosensors identify biological signals. Interest in using nanostructures in biotechnological systems has increased with the growing accessibility of nanostructures with controllable characteristics in the nanometer size range. Nanostructures have novel and distinctive qualities, such as the ability to precisely manipulate their structure and adapt them to the needs of biological systems. These nanostructures enable novel hybrid structures for application, including biosensing and imaging, and may be utilized for imaging and biosensing and incorporated in biocompatible materials. Biomolecules and non-biologically produced molecular species are combined in bioconjugate chemistry for specialised usage in various applications, such as biosensing, bioimaging, or targeted medication administration. Higher quantum efficiencies, larger dispersion or absorbance cross-sections, improved optical activity throughout biocompatible wavelengths, and better chemical or photochemical endurance are only a few of the exceptional qualities of nanostructures. Nanoparticles are classified based on their size, morphology, and physical and chemical properties [20].

Carbon-Based Nanoparticles

Carbon-based nanomaterials include fullerenes, carbon nanotubes, graphite and its derivatives, graphene oxide, nanodiamonds, and carbon-based quantum dots. These materials have attracted interest in many fields, including biological applications, due to their remarkable mechanical, electrical, thermal, optical, and chemical properties and unusual structural size. Although drug and gene delivery is still in its infancy, research into carbon-based nanomaterials for use in biomedical applications has garnered a lot of interest. Carbon-based nanomaterials, particularly graphene oxide nanosheets, are attracting attention in the emerging field of medicine and gene delivery. These nanosheets can increase therapy accuracy and reduce adverse effects by delivering medications to specific cancer cells. They are promising candidates for propelling biological uses in tailored and effective treatments because of their distinct characteristics. Its utilization in the conveyance of medications and qualities is a hot issue. Bioimaging many imaging applications have long been seen as carbon-based materials, for example, single photon emanation and mechanized positron outflow tomography (PET) and fluorescence imaging (FL). Single photon outflow is an atomic imaging method utilizing gamma-discharging radiotracers. It records the emitted photons and creates precise three-dimensional images of internal structures for medical diagnostics, particularly in neurology and cardiology. Utilizing positron-emitting radiotracers, the medical imaging procedure known as positron emission tomography allows for the visualization of metabolic processes. It helps diagnose and monitor diseases by providing functional, detailed images. Fluorescence imaging is a painless method utilizing fluorescent colours to envision and concentrate on cell and subatomic cycles. It upgrades biomedical examination and diagnostics by giving ongoing, high-goal imaging [21]. There is a fear that carbon nanotubes might lead to asbestos-related disorders like mesothelioma (pleural cancer) or lung interstitial fibrosis because they have an aspect ratio of length to width with asbestos fibres [22].

Ceramic Nanoparticles

One of the most prevalent materials in daily life, ceramic materials have a special position in people's work and daily lives that few other materials can fill. The early ceramics that people manufacture have flaws of fragility and limited flexibility because of their delicate surface [23]. Bone repair nanoscale bio-ceramics can bind to bone tissue, mirror its nanocomposition and nanoscale characteristics, and promote osseointegration and bone development at the bone-implant interface, as well as the osteoconductive and osteoinductive capabilities of the implant, in comparison to their micro-sized conventional counterparts [24]. Multiscale hybridisation, due to the significance placed on the longevity and dependability of individual structural components and the intricacy of different loading rates on the structure as a whole, i.e., the aeroplane and aeronautical structures, gets a lot of attention. Due to their high strength, excellent corrosion resistance, low weight, cheap cost, and simplicity of manufacturing, thin-walled structures (TWS) made of fibre-reinforced polymers (FWPs) are often employed in construction [25]. Reticuloendothelial system inflammation and oxidative stress are common side effects of ceramic nanoparticles. Cytotoxic activity is experienced by the heart, liver, and lungs due to inflammation. Due to their larger surface areas, the majority of ceramic nanoparticles are significantly more damaging to the linked cell. When ceramic nanoparticles produce free radicals, they often generate oxidative stress, which can lead to genotoxicity, inflammation, and cell death [26].

Metal Nanoparticles

Although a distinct physical mechanism creates them, metal nanoparticles have intriguing linear optical features that are conceptually similar to molecular systems. Particle shape and interparticle interaction are crucial for absorbing plasmon resonance, giving metal nanoparticles a distinctive colour [27]. Noble metal nanoparticles have several biological uses (Ag, Au, and Pt), including anticancer, improved radiotherapy, medication administration, thermal ablation, antibacterial, diagnostic tests, antifungal, gene transfer, and many others [28]. The study of anticancer agents shifted from organic molecules to embrace metallo-pharmaceuticals. Numerous platinum and platinum-based substances, such as carboplatin and oxaliplatin, have received approval as anticancer medications. However, several disadvantages of medications based on platinum have been documented, demonstrating their therapeutic efficacy [29]. Antifungal metal nanostructures called silver nanoparticles (AgNPs) exist. AgNPs have demonstrated antibacterial activity against fungi and bacteria; nevertheless, synthesising AgNPs can produce harmful waste [30]. Metal nanoparticles were used at different doses to test their in vitro antifungal effectiveness. Using silver nanoparticles at a dosage of 15 mg L-1 resulted in the greatest suppression of fungus hyphal development. The efficacy of mixing silver and copper nanoparticles was also evaluated. Microscopical analysis showed that fungal hyphae and conidia were damaged by nanoparticles [22]. Magnetic nanoparticles (MNPs) that enter the body alter inflammatory cytokines. Nickel oxide nanoparticles (NiONPs) decreased IL-4 and IL-10 but raised IL-1 and IL-6, indicating a shift towards pro-inflammatory cytokines. MNP-induced liver dysfunction results in structural alterations to the liver. MNPs cause inflammation, which potentially changes liver function. A considerable drop in total bilirubin and increased alkaline phosphatase (ALP) and aspartate aminotransferase (AST) markers in the blood serum suggested liver damage. The damage was mostly seen as structural alterations in the liver that led to metabolic dysfunction [31].

Semiconductor Nanoparticles

A wide range of various substances are used to create semiconductor nanocrystals (NCs). According to the periodic table, the divisions under which these elements are produced are often referred to as group II-VI, III-V, or IV-VI semiconductor NCs. GaN, GaP, GaAs, InP, and InAs, for instance, are III-V semiconductors, while ZnO, ZnS, CdS, CdSe, and CdTe are II-VI semiconductors [4]. Despite being in the research stage, semiconductor nanomaterials and devices have the potential for use in a variety of sectors, including solar cells, nanoscale electronics, light-emitting nanodevices, laser technology, waveguides, chemicals, and biosensors [32]. Laser technology nanotechnology has the potential to significantly boost solar energy production and storage while assisting in overcoming current efficiency barriers. Light absorption, energy conversion, and heat storage and transfer have all been demonstrated to be enhanced by nanoparticles and nanostructures [33].

Biosensors: These materials are highly desirable in the biosensor market based on chemical stability, photon excitation/light conversion, and a large surface area-to-volume ratio. There are promising results for detecting cancer cells or viruses, while most biosensors are employed for glucose (H2O2, uric acid). Recent developments suggest that metal oxide semiconductor (MOS) has a bright future in applications, including skin bioelectronics and brain interfacing [34].

Polymeric Nanoparticles

Small particles known as polymeric nanoparticles can have active chemical surface adsorbed within their polymeric core and range in size from 1 to 1000 nm. The term "nanoparticles," which includes nanospheres and nanocapsules, is defined by their morphological structures [35].

Polymeric nanoparticles in the delivery of vaccines: Polymeric micro- or nanoparticles have been used to transport genes, particularly in developing vaccinations (such as DNA vaccines). Additionally, gene therapy has demonstrated a tremendous potential to benefit patients with various medical situations [36]. Polymer nanoparticles (nanomedicine) for therapeutic applications have diameters ranging from a few nanometers to 1000 nm and various shapes and morphologies. These are also small solid and colloidal particles. For technical advancements in medication delivery and other healthcare uses, it is a novel type of material created by nanotechnology [37]. Polymeric therapeutics have had nanomedicines that offer several advantages for treating infectious diseases, such as a specific medication-release mechanism, the ability to keep the drug concentration to feed a therapeutic period for a desired amount of time, biocompatibility for low immunogenicity, and decreased drug toxicity, which leads to an increase in the therapeutic efficacy for the incorporated drug.

Lipid Nanoparticles

The most sophisticated non-viral gene delivery technique currently accessible is lipid nanoparticles. Lipid nanoparticles efficiently and securely transport nucleic acids, reducing a major barrier to creating and using genetic therapeutics. Genetic medicine has several uses, including gene editing, quick vaccine creation, immunological oncology, and the treatment of uncommon genetic and heritable illnesses, often constrained by ineffective nucleic acid delivery [38]. Lipid nanoparticles transporting messenger RNA (mRNA) emerge as novel therapeutic agents for preventing and treating various illnesses, reflecting mRNA's evolution. Delivery mechanisms must be trustworthy, efficient, and long-lasting for mRNA to operate in vivo. Additionally, they must allow for cellular absorption and mRNA release while safeguarding the DNA from deterioration. Lipid nanoparticle-mRNA vaccinations are currently utilised in clinical trials to treat coronavirus disease 2019 (COVID-19), an important step for mRNA therapeutics. Lipid nanoparticles are being used to efficiently deliver mRNA into the clinic [39].

Cancer-causing lipid nanoparticles: The most prevalent cancer in women is breast carcinoma, and its incidence rate has been gradually rising over time. Effective breast cancer chemotherapies are significantly hampered by rapid clearance, systemic toxicity, insufficient medication concentrations near the tumour, and adverse effects. Solid lipid nanoparticles (SLNs) can circumvent current chemotherapeutic limitations and the problems associated with standard chemotherapy and multidrug resistance (MDR) in the treatment of breast cancer [40].

Future

Nanotechnology, a promising enabling technology of the 21st century, has grown since its inception in the 1980s. It contributes to various fields, including natural sciences, engineering, materials science, medicine, agriculture, and information or communications technologies. Nanoparticles have the potential to revolutionize a wide range of industries, including pharmaceuticals (for specific drug delivery and imaging), hardware (faster processors, efficient energy storage), and environmental assembly. Their unique nanoscale characteristics assure remarkable advancements, promoting progress and addressing intricate issues in various domains in the future. As society faces health, energy, climate, and environmental challenges, new technologies are needed to address these issues. In November 2020, the Global Network for Sustainability Nanotechnology (N4SNano) was hosted by the Waterloo Institute for Nanotechnology and was founded because of a digital international workshop for nanotechnology on a sustainable future. With the help of this network, governments, policymakers, and scientists will be able to embrace technological solutions to contemporary issues more easily [41]. Since the control of the underlying molecular structure enables the control of macroscopic physical and chemical properties, the consideration of individual molecules or linking groups of molecules with the large macroscopic properties of a substance or device becomes critical. Materials and tools having an elevated degree of specificity for subatomic (i.e., molecule) connections to the body are used in applications to medicine or physiology. It might lead to the development of cellular and tissue-specific medical treatments to enhance therapeutic advantages and avoid adverse effects [20]. Table 1 details the findings of the articles included in the review. 

**Table 1:** Characteristics of articles included in the review HCV: hepatitis C vaccine; GNP: gold nanoparticle

Authors	Year	Findings
Arca-Lafuente et al. [1]	2020	The traditional HCV diagnosis procedure starts with a serological test and then moves on to a nucleic acid test.
Abel et al. [2]	2023	There is yet no medicine that can effectively and safely cure obesity.
Fernandez [3]	2007	Advances in diagnostic, regenerative, and pharmaceutical therapeutics will be made using nanotechnology approaches, nanomedicine, and nanotechnology.
Núñez-Toldrà et al. [4]	2020	Flexoelectricity is involved in bone remodelling in at least two separate ways: as an electro-stimulant for osteoblasts' bone-building activity and as an apoptotic trigger for the repair process.
Piantanida et al. [5]	2019	The hydrogel network can have its structural and functional features expanded, improving the hydrogel's ability to resemble real tissues.
Hulla et al. [6]	2015	Despite the fact that humans have been exposed to nanoparticles throughout history, during the Industrial Revolution, this exposure drastically increased.
Chen and Feng [7]	2022	Physical techniques that may be used in the future to improve the effectiveness of GNPs' distribution were also discussed.
Bucciarelli et al. [8]	2021	This procedure involves boiling the silk cocoons for a certain amount of time in an alkali bath.
Gabbay Alves et al. [9]	2017	A Box-Behnken factorial design was used for the microencapsulation testing.
McNeil [10]	2005	For biologists, nanoparticles' tiny size, surface tolerability, increased solubility, and multifunctionality enable a wide range of new study opportunities.
Sacha [11]	2005	Raman spectroscopy, MRI, X-ray diffraction, mass spectrometry, and some references to more thorough therapies.
De Paula et al. [12]	2018	Examine the impact of the coating on banana firmness, weight loss, pH, titratable acidity, and soluble solids.
Vaneev et al. [13]	2021	The amount of medication that reaches the internal ocular tissues when supplied as eye drops is less than 5%.
Rode et al. [14]	2018	The amount of medication supplied in eye drops that reaches the internal ocular tissues is less than 5% of the overall dosage.
Bartneck [15]	2017	Recent progress in cancer immunotherapy uses adaptive leukocytes to manipulate innate immune cells, notably macrophages, for cancer cell destruction.
Sacha and Varona [16]	2013	Future thoughts on the creation of nanocomputing hardware that can enhance applications based on artificial intelligence.
Yuwen et al. [17]	2023	Nucleic acid and protein detection are the mainstays of viral detection.
Chen et al. [18]	2018	DNA nanotechnology may be useful in achieving the aims of early cancer detection and prompt cancer treatment.
Narayanan and Abraham [19]	2022	This article covers the assembly of DNA nanostructures utilising novel (but promising) methods, including quadruplexes, kissing loops, and i-motifs.
Silva [23]	2004	Applications to medicine and physiology involve materials and equipment with a high degree of specificity for subcellular (i.e., molecule) interactions with the body.

## Conclusions

Nanotechnology is a powerful tool capable of transforming many fields and is a cutting-edge field at the intersection of science, engineering, and technology. At the nanoscale, where materials exhibit unique properties and behaviours, this involves manipulating and controlling matter. Scientists and researchers have opened up possibilities using the principles of nanotechnology that have led to significant advances in fields such as medicine, electronics, energy, and materials science. Nanotechnology in healthcare and medicine is one of its most impressive features. The field of medicine known as nanomedicine offers new approaches to drug delivery, diagnosis, and therapy. Medicines can be more effective by carefully targeting diseased cells or tissues with nanoparticles and minimizing adverse effects. Nanosensors and nanodevices can also monitor and detect diseases at an early stage, enabling rapid intervention. In addition, nanotechnology has helped create advanced imaging techniques that provide medical diagnosis with unprecedented sensitivity and resolution. Nanotechnology has completely changed how we manufacture and develop electronic devices in the electronics industry. Faster, more compact, and efficient devices result from the miniaturization of components. Next-generation transistors, batteries, and displays can benefit significantly from the extraordinary electrical properties of nanoscale materials such as carbon nanotubes and graphene.

In addition, nanotechnology enables the creation of flexible and portable electronics, creating new possibilities for consumer electronics and medical monitoring devices. Another area where nanotechnology has made great strides is the energy sector. Solar cells are reinforced with nanomaterials that improve the efficiency of sunlight conversion. Energy storage systems such as batteries and supercapacitors could benefit from nanotechnology, increasing capacity and durability. In addition, nanotechnology is used to create more efficient fuel cell catalysts that improve energy conversion processes. Thanks to nanotechnology, the development of innovative materials with improved properties has dramatically helped the field of materials science. Compared to traditional materials, nanocomposites made of a matrix with embedded nanoparticles have higher strength, conductivity, and durability. Many industries use these materials, including aerospace, automotive, and construction. In addition, nanotechnology enables self-cleaning surfaces, non-reflective coatings, and superhydrophobic materials, which offer new possibilities for various applications. Challenges facing nanotechnology include the potential toxicity of nanoparticles, environmental effects, and ethical issues. Mass production is hindered by the high cost of manufacturing precise nanoscale structures. Public fear and regulatory restrictions also hinder wider adoption. Ethical development and innovation must work hand in hand to maximize nanotechnology and its benefits and minimize its risks.
